# A Developmental Model for Branching Morphogenesis of Lake Cress Compound Leaf

**DOI:** 10.1371/journal.pone.0111615

**Published:** 2014-11-06

**Authors:** Akiko Nakamasu, Hokuto Nakayama, Naomi Nakayama, Nobuhiko J. Suematsu, Seisuke Kimura

**Affiliations:** 1 Department of Bioresource and Environmental Sciences Faculty of Life Sciences, Kyoto Sangyo University, Kyoto, Japan; 2 Meiji Institute for Advanced Study of Mathematical Sciences, Meiji University, Tokyo, Japan; 3 Institute of Molecular Plant Sciences, University of Edinburgh, Edinburgh, Lothian, United Kingdom; 4 Graduate School of Advanced Mathematical Sciences, Meiji University, Tokyo, Japan; The University of Tokyo, Japan

## Abstract

Lake cress, *Rorippa aquatica* (Brassicaceae), is a semi-aquatic plant that exhibits a variety of leaf shapes, from simple leaves to highly branched compound leaves, depending on the environment. Leaf shape can vary within a single plant, suggesting that the variation can be explained by a simple model. In order to simulate the branched structure in the compound leaves of *R*. *aquatica,* we implemented reaction-diffusion (RD) patterning onto a theoretical framework that had been developed for serration distribution in the leaves of *Arabidopsis thaliana,* with the modification of the one-dimensional reaction-diffusion domain being deformed with the spatial periodicity of the RD pattern while expanding. This simple method using an iterative pattern could create regular and nested branching patterns. Subsequently, we verified the plausibility of our theoretical model by comparing it with the experimentally observed branching patterns. The results suggested that our model successfully predicted both the qualitative and quantitative aspects of the timing and positioning of branching in growing *R*. *aquatica* leaves.

## Introduction

Morphogenesis of multi-cellular organisms requires coordination of growth and developmental pattern formation (i.e., temporal and spatial specification of morphogenic fates). Morphogenesis accompanied by continuous growth requires stable positional information in order to make well-proportioned shapes. From the structure of lungs to the branching of trees, nearly evenly spaced branches represent a major type of morphogenic process and various mathematical models have been applied to explain each phenomenon [1], [2].

Branching morphogenesis in plants is often accompanied by growth. One example is compound leaf development. Although plant leaves exhibit striking diversity in their shapes, they can be roughly classified into two classes: simple and compound. Simple leaves have a single blade, whereas compound leaves have multiple blade units. In both types of leaves, a leaf begins as a small bulge, called a leaf primordium, at the tip of the shoot apex. The leaf primordium grows rapidly by cell division and cell expansion, eventually forming the mature leaf shape. In the case of compound leaves, new leaflet primordia emerge iteratively on a growing leaf primordium [3], [4]. Recently, a laser ablation experiment on *Eschscholzia californica* leaf primordia revealed that a constant-spacing regulatory mechanism governs leaflet initiation sites [5].

Some geometric features of leaf shapes have been theoretically investigated [6]. The theoretical understanding of leaf shape determination has recently dramatically improved [7], [8], [9]. For example, Bilsbrough et al. (2011) explained the mechanism underlying the formation of a simple leaf with serrations on its margin by using a mathematical model that utilized a repeated iterative pattern. It has been suggested that the framework of this serration model could also explain the morphogenesis of more complex leaf shapes [8]. The spatially periodic pattern was simulated using the polar auxin transport (PAT) model, which has been widely used to explain various aspects of plant morphogenesis, including phyllotaxis, leaf vascular patterning, and root development [10], [11], [12], [13]. In the leaf morphogenesis, PAT was caused by interactions of the growth-promoting phytohormone auxin, PIN-FORMED1 (PIN1: auxin efflux carrier protein) [14], and CUP-SHAPED COTYLEDON2 (CUC2: transcription factor that negatively regulates local growth rate) [15].

**Figure 1:**
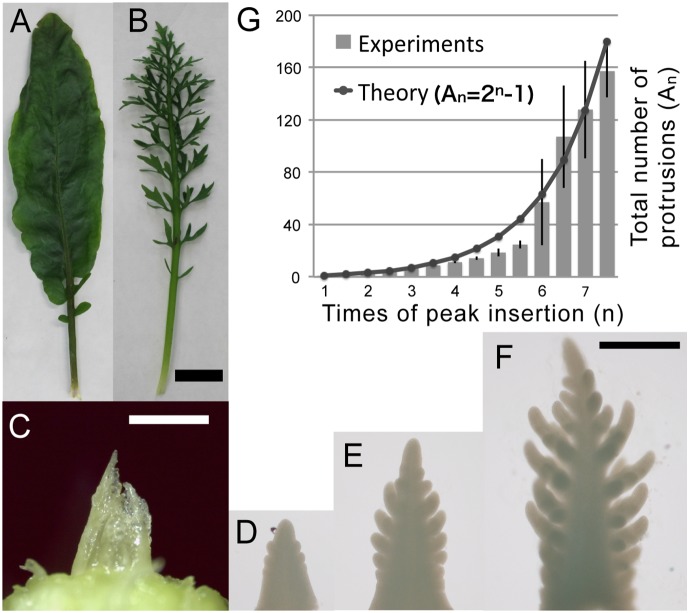
Morphogenesis of *Rorippa aquatica* leaves. A, B: Mature leaf morphology of the simple leaf that was developed at 30°C (A) and the highly branched compound leaf that was developed at 20°C (B). Scale bar: 1 cm. C: Dissected shoot apex of a plant grown at 20°C, showing the nested group of leaf primordia with indented blade. D–F: Dissected primordial of a plant grown at 20°C for about 2 months. Each primodium has the 32th (D), 35th (E), and 39th (F) leaf primordium from the oldest (i.e. outermost) leaf of a plant. The larger leaf position numbers indicate younger leaves. Scale bar: 1 mm (C) and 200 µm (D–F). G: Comparison of the total number of leaflet primordial between experimentally observed and the theoretically estimated value.

We studied the North American lake cress (*Rorippa aquatica*), which is a semi-aquatic member of the family Brassicaceae, as a model to explain leaf diversity. *R*. *aquatica* changes the shape of its leaves depending on the growth conditions (e.g., underwater submergence and temperature) [16]. Such morphological plasticity is called heterophylly. The leaf shape can range from simple elliptical to a complex, highly branched compound morphology (Fig. 1A, B). Intermediate forms have also been observed. This plant is a theoretically good model to study leaf morphogenesis since such variation in the leaf shape is observed even within a single plant. There may be a common morphological mechanism that produces the various leaf-shapes in heterophylly. If so, it can be explained by a simple model.

Here, we built the model to explain the morphogenesis of the highly branched compound leaves in *R*. *aquatica*. Instead of PAT, we utilized RD based patterning [17], [18], [19] for our model because it is often employed to explain spatial pattern formation in biological systems, such as the pigmenting stripe pattern of marine angelfish [20]. The pattern behavior on a growing domain is well studied and known to be easily controlled [21]. Diffusion-driven instability, which was suggested by Alan Turing in 1952, can create stationary periodic patterns called Turing patterns. They are known to cause the insertion or splitting of reactant peaks to maintain equal intervals when placed on an expanding domain [21], [22].

Our model was successful in replicating the growth and branching events in the development of *R*. *aquatica* compound leaves. It was able to predict the qualitative aspects, such as the positioning, direction, and order of branching, as well as the quantitative aspects, including the increase in the number of branches.

## Results and Discussion

### Morphological observation

*R*. *aquatica* develops smooth-margin simple leaves when grown at 30°C, and compound leaves of a multi-order branched structure when grown at 20°C (Fig. 1A, B). The simple leaf has iterative hydathodes on its margin, which are generally seen at the tip of serrations in *A*. *thaliana*. The leaves at the earliest stages of development at the shoot apex were dissected and used to examine the branching pattern formation (Fig. 1C). A shoot apex contains leaf primordia at different stages of morphogenesis (Fig. 1D–F). By the observation of dissected shoot apex and from time-lapse movies [Nakayama et al. in preparation] the branched structure of the compound leaf formed through repetitive additions of protrusions that are future leaflets (i.e., leaflet primordia). The number of leaflets increased gradually (Fig. 1D–F). First, primary leaflets appeared on both sides of a leaf primordium (Fig. 1D), and secondary leaflets emerged on the primary leaflets after some elongation (Fig. 1E, F). The increase in the total number of leaflets of all categories (i.e., terminal, primary, secondary, tertiary) was plotted as a progression of the primary leaflet formation (Fig. 1G). The dynamics of repetitive leaflet initiations prompted us to consider that a spatially periodic pattern occurs during compound leaf development in *R*. *aquatica*.

The total number of leaflets reached a plateau when the leaf became approximately 4 mm in height. At this point, the number of primary leaflets also reached a plateau. Because the number of leaflets on the primary leaflet of mature leaves is almost the same as the number of leaflet primordia on primary leaflets of immature leaves as described later, it is suggested that the branched structures of both mature and immature leaves are geometrically similar. This means that branching is fixed after that specific stage, and the duration of leaflet formation seems constant and determined. From time-lapse recordings of developing leaves, it was observed that new protrusions emerge in the recently elongated regions of a leaf, which exist on the both sides of the tip and the base of the first primary leaflets [Nakayama et al. in preparation].

### Modeling for branched structure of compound leaves

**Figure 2:**
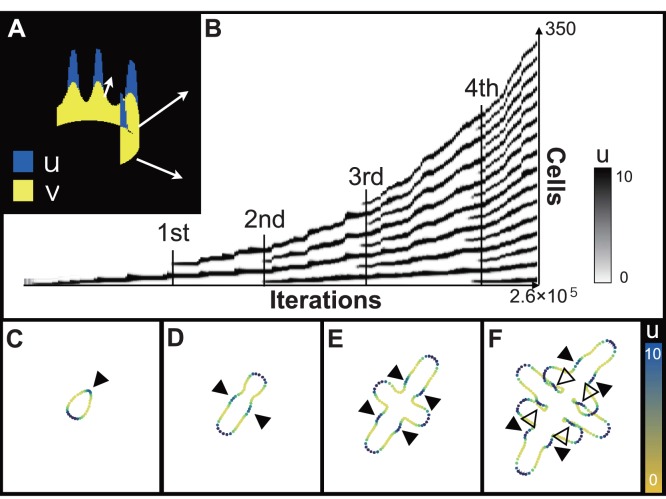
Spatiotemporal plot and growth profiles for the BPM rings by pattern dependent expansion. A: Schematic of the modeling. B: Spatiotemporal plot for peak doubling by insertion. The value of reactant 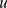 is represented by the gray scale. Each panel shows the first (C), second (D), third (E), and fourth (F) insertion. Each point indicate the middle point of segmented cell, then the color of points indicate the value of reactant *u*. Solid arrowheads indicate the points of peak insertion, and empty arrowheads are points of side branch generation.

In order to explain the branching dynamics in *R*. *aquatica* leaves, we utilized the framework of a previously developed mathematical model [9] (Fig. 2A). The leaf margin was regarded as a one-dimensional reaction-diffusion domain, which was composed of segmented lines simulating cells. The leaf margin was deformed based on the spatially periodic pattern using the boundary propagation method (BPM), which abstractly achieves this geometric deformation [23]. Briefly, the growth direction of the connection point was vector sum of the normal unit vector of adjacent cells and the growth rate was proportional to the average concentration of reactant in adjacent cells. Segments over a certain length were divided into two daughter cells, which had the reactants state of mother cell.

The growth profile of the BPM ring and the spatiotemporal pattern of the reactant peaks therein are shown in Figure 2. To support the experimental observations, in which new branches were inserted into newly generated gaps between two leaflets, we calculated the RD pattern formation using the parameters that showed insertions of reactant peaks. Expansion at the position of reactant peak makes the peak unstable then it generally induces the peak splitting. However, in appropriate parameter region, regular peak doubling by insertion can be generated (Fig. 2B, Movie S1). For easy selection of pattern behavior on growing domain, the linear activator-inhibitor model was used.

As an initial condition, six cells that had random values of reactants *u* and *v* were connected as a ring. The ring grew in one direction based on the location of the first-emerged reactant peak (represented blue in; Fig. 2C–F, Movie S1) and subsequently, the first insertion was generated (Fig. 2C, Movie S1). The second peak emerged on the opposite side of the first peak. When we regarded either of them as a terminal leaflet, the ring was stretched toward each peak to make the main axis (i.e., the proximodistal axis) of leaf growth. Next, tertiary peaks were formed in the center of intervals, which become wider between the first and second peaks, resulting in two secondary axes that corresponded to the primary leaflets (Fig. 2D, Movie S1). As the reactant peaks were inserted in order, new secondary axes joined four by four on the recently extended region of the main axis (Fig. 2E, Movie S1). In addition, ternary axes, corresponding to the secondary leaflets, were generated on the extended region of the secondary axes (Fig. 2F, Movie S1).

The simulated branching dynamics recapitulate the qualitative aspects of the morphogenesis of compound leaves in *R*. *aquatica*. Hence, the developmental model for indented simple leaves [9] is also applicable to the morphogenesis of branched compound leaves. The behaviors of spatially periodic patterns on uniform growing domain have been well studied and analyzed. On the other hand, the study about the behaviors of the patterns on reactant-dependent growing domain is still limited especially in the case of peak insertion. Here, we show that a linear RD pattern can be reduplicated by regular insertion using appropriate parameters. Whatever the mechanism governing the periodic pattern, this nested branched pattern was generated by the simple rule of a continuous growth dependent on spatially periodic pattern.

### Comparison between the simulation and actual-leaf branching dynamics

**Figure 3:**
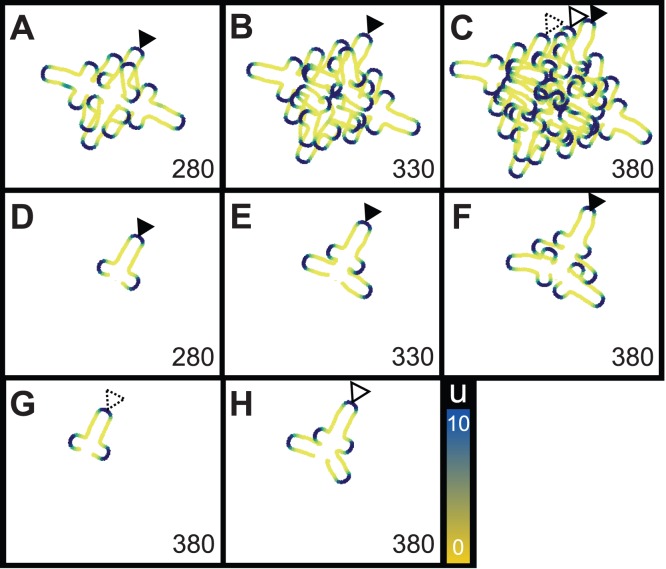
Simulations of leaf primordia and branches. Each panel shows the simulated whole leaves (A–C) and primary leaflets (D–H). The simulated branches were crossover. Each branch was independently formed nested regular branches. The inserted number shows the time of iterative calculations (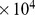), and the arrowheads indicate each leaflet; filled, flamed, and dotted arrow heads represent the first, second, and third primary leaflet respectively.

Even with the distinctive character, the snapshot of shapes of organisms can be simulated by various models. Therefore, it is important to compare the spatial-temporal profile of developmental events to reduce the degree of freedom. To test the plausibility of our model, we compared the actual morphogenesis of *R*. *aquatica* compound leaves to the branched pattern generated by our simulation. Although the simulated branches overlapped and had tangled crossovers, regular and spatiotemporally nested branches were generated as the iterative calculation progressed (Fig. 3, Movie S2). Each branch was formed by a closed ring, and branches were thus independent of each other (Fig. 3F–H and Movie S2). It should be noted that since the actual structure of the leaf primordium varies in three-dimensions, the crossovers of each branch are irrelevant.

When we expressed the number of terminal and primary leaflets where 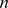 times insertion had occurred as 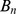, we observed an increase in the number of the primary leaflets over time. At first, only the terminal leaflets were produced by first insertion, and thus the number of total leaflets was given by 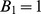. For every 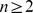
_,_ the addition of primary leaflets led to the formula 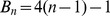, thereby ensuring that the additions were proportional to the times of the events. Therefore, we expressed the morphogenetic stage as the number of primary leaflets and placed it on the horizontal axis (Fig. 1G).

When we considered the nature of the peak doubling, the estimation of the total number of leaflets 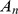 was given by the simple formula 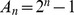, which was obtained from the subtraction of the one peak corresponding to the base of leaf primordium from 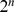
_,_ the total number of the peaks. The theoretical estimation was shown as a solid line marked by squares, and the total number of leaflets from the experimentally observed half primordium was shown as a bar with standard error (Fig. 1G). Despite slight deviations, similar increments were observed in both data sets, suggesting the plausibility of our model.

Next, we compared the generation of secondary leaflets on each primary leaflet. When we put the estimation of the number of secondary leaflets on the *i* ^th^ newest primary leaflet, 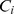, and gave 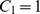 could be represented as 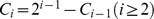. This simple recurrence formula stems from the characteristics of branches being nested and having a regular branching rule that shown by this model (Fig. 3, Movie S2).

The total number of leaflets mentioned above could be represented by the summation of individual leaflets. Therefore, the total numbers of leaflets on *n* times inserted leaf primordium 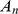
s which contain a terminal leaflet and 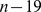 ^th^ newest two first primary leaflets and following four primary leaflets were given using 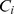
s as 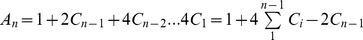 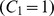 and thus 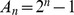.

**Figure 4:**
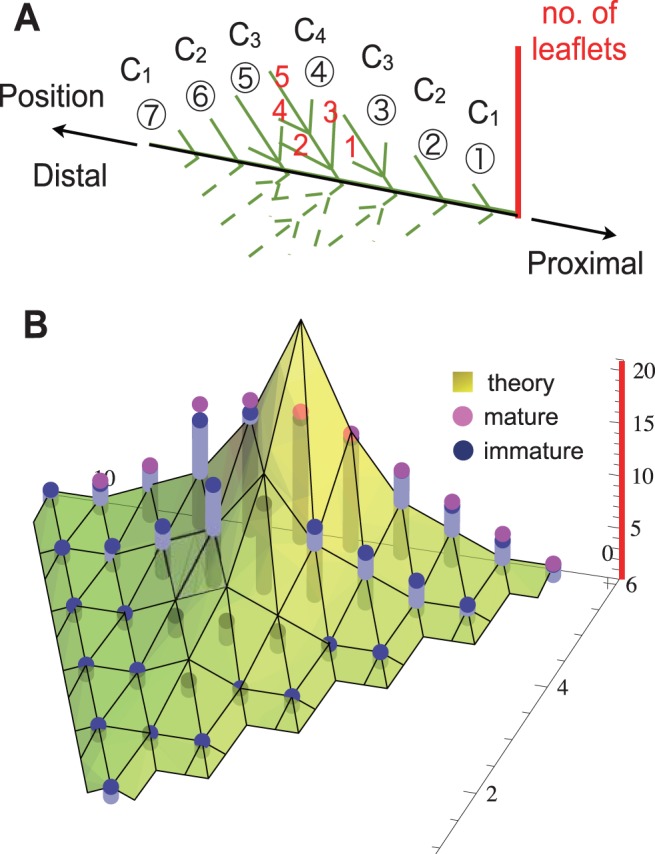
The numbers of leaflet primordia of each primary leaflet. A: Schematic of the branched structure of one half of a *R*. *aquatica* compound leaf. Circled numbers indicate the positions of primary leaflet (the horizontal axis in B), and theoretically derived recurrence formulas of each primary leaflet are shown by 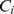. The red numbers represent the numbers of leaflets formed on the 4^th^ primary leaflet (the vertical axis in B). B: A comparison between the experimentally observed data in actual plants and theoretically estimated numbers derived from mathematical formulae of leaflet on each primary leaflet. The magenta dots show the data from mature leaves. The number of leaflets at each stage was plotted as aligned at the center. The theoretical estimations are represented on a yellow planar graph, and the actual data in developing leaves as blue dots with columns.

Since the leaf could be regarded symmetrical along the proximodistal axis, we considered a half of the branched leaf for comparison. As in Figure 4A, we placed the positions of primary leaflets on the half leaf and the numbers of leaflets on each primary leaflet on the horizontal and vertical axis, respectively (Fig. 4B). The spatial branching structure of mature leaves (expanded leaves larger than 5 cm in length at leaf number 10–15) were indicated as magenta dots in Fig. 4B. For immature leaves, we grouped the graphs of blue column corresponding to each primordium at different morphogenic stages. Furthermore, values of 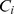 against the positions of the primary leaflets were plotted on the planar graph (Fig. 4B).

The analysis that focused on the branching of each primary leaflet showed consistency between the theoretical estimation from the mathematical formula and empirical data in actual plants, although some discrepancies were observed. In the simulation, the number of total leaflets increased from the centrally located primary leaflet, since it was generated first (earliest). On the other hand, the actual location of primary leaflets with the highest number of branches tended to be toward the apical side, rather than the center. Furthermore, the natural leaflet distributions varied mildly compared to the peaked theoretical values.

### Branch formation by expansion inhibition

**Figure 5:**
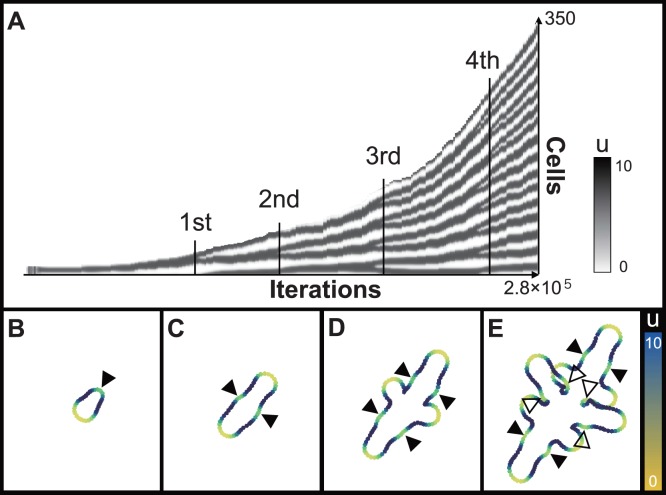
Spatiotemporal plot and growth profiles for the BPM rings by Expansion inhibition. A: Spatiotemporal plot for peak doubling by splitting. The value of reactant 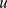 is represented by the gray scale. Each panel shows the first (B), second (C), third (D), and fourth (E) splitting. Each point indicate the middle point of segmented cell, then the color of points indicate the value of reactant *u*. Solid arrowheads indicate the points of peak splitting, and empty arrowheads are points of side branch generation.

Recently, Vlad *et al.* reported that cell proliferation in the sinus region was inhibited in a gene expression pattern dependent manner at the formation of a compound leaf in *Cardamine hirsuta* [24]. Therefore we attempted a method that was opposite case to that described in the previous sections. The leaf margin was expanded constantly then the expansion was inhibited based on the spatially periodic pattern. As a result, fundamentally equal branched pattern and branching process were observed (Fig. 5B–E, Movie S3), even in the case of expansion inhibition. Because the region without the reactant peaks bulged outward, the branching was generated by repetitive splittings of the pattern (Fig. 5A, Movie S3). This is simply the reverse case of the insertion of Turing-pattern behavior on growing domain [21]. Insertion of peak emerges at the region where the activator is deficient. Thus, it is important to keep the distance between two successive peaks of activators relatively large while domain growth to make insertion happens. To make splitting happen, opposite situation is needed.

Our simulation created symmetrical and regular branched structures, and the timing of peak-doubling was synchronous; however, the actual *R*. *aquatica* leaf undergoes asynchronous branching. The asynchronous branching can be caused, even in the equal interval pattern, by the introduction of anisotropic growth. In such cases, it is difficult to accurately estimate the increase in the number of leaflets by the methods described in this work. In addition, we assumed interactions among branches in this model; without the interactions, the same results would be calculated using certain timings of branching. Therefore, in order to confirm the biological significance of this model, the existence of interactions among branches should be tested. Recently, the presence of such interactions between leaf tip and leaflet of the compound leaves was shown by a laser ablation experiment in *E*. *californica* [5].

The model reported here may require further verification; however, it has the potential to simulate the full spectrum of the morphogenic gradient of leaf shapes in *R*. *aquatica*. It has already been led to a specific new hypothesis to be tested experimentally that will likely reveal new insight into the mechanism behind the diversity of leaf shapes. The molecular mechanisms of compound leaf development have been studied in model plants, such as tomatoes (*Solanum lycoperisicum*) and *Cardamine hirsuta* [3], [25], and the differences in the timing and positioning of maturation within the primordia have been related to the species-to-species differences in the direction and position of leaflet initiation [4], [26]. The molecular basis of the reactants used in this RD based model has not yet been determined. We would like to determine the molecular aspect of this mechanism in the future.

## Materials and Methods

### Plant materials and growth condition

Plants were grown in soil for 1.5–2 months in a growth chamber under a continuous light condition with the temperature set at 20°C at a photo flux density of 70 µmol m^−2^ s^−1^.

### Dissection and image acquisition

To investigate the spacing of the branch formation, 12 shoot apices were mounted on a glass slide and dissected under a stereoscopic microscope (LEICA 8AP0) with a LAS EZ digital camera using forceps. The shoot apices were fixed in FAA (4% formaldehyde, 5% acetic acid, and 50% ethanol) and were washed using phosphate buffered saline (PBS) before dissection. Images were captured by a digital camera (Nikon DS-Ri1) mounted on an up-light light microscope (Nikon ECLIPSE 80i).

### Simulation

The simulation was performed according to the method described by Bilsbrough et al. (2011). Leaf margin, regarded as a one-dimensional reaction-diffusion domain, was expanded based on the spatially periodic pattern which was generated by the RD model as described in [20]. The differential equations used in the simulation were as follows:
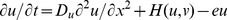


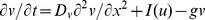


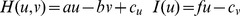


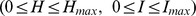



The linear activator-inhibitor model using two variables, an activator “
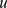
” and an inhibitor “
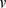
” were chosen for their flexibility of pattern-behavior selection. The parameters used in the simulation were: 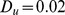, 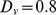, 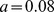, 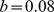, 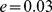, 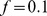, 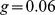, 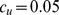, 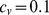, 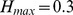, 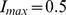 in case of pattern dependent expansion then 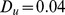, 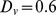, 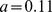, 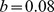, 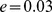, 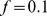, 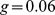, _mi_, 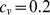, 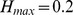, 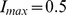 in the latter case that is expansion inhibition. They were selected to lead stable spatially periodic insertions or splitting of reactant peaks on the expanding reaction-diffusion domain. The model parameters were searched based on empirical knowledge for 2D simulation of Turing pattern by linear model. The parameters making spot and net pattern in 2D tend to form insertions and splittings on growing domain, respectively. Leaf margin was simulated using the boundary propagation method [23], i.e., the propagation of the leaf margin in space over time. This propagation was performed iteratively by updating connection points of arbitrary segments regarded as a cell. Connection points (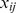, 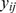) of adjacent cells 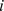 and 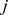 were displaced at velocities 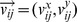, which were activated by reactant u as 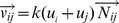 or inhibited by 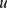 as 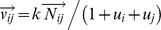. 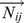 were the propagation directions, and the summation of normal vectors of the margin of cells 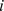, 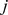 pointed outward. 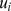, 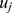 indicate the amount of reactant 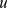 of cell 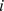, 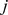 respectively. The 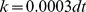 in the case of pattern dependent expansion, then the 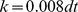 in the case of expansion inhibition. A cell divides into two daughter cells when its length exceeds a threshold by updating positions (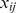, 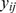). The two daughter cells inherited the reactant state of their mother cell.

## Supporting Information

Movie S1 Branch formation by pattern dependent expansion. Pattern dependent expansion of the ring was simulated. Left panel shows the growth profile of the ring. First, second, and third insertions are marked by arrows. Right panel shows the spatiotemporal plot of growing reaction-diffusion domain. Pattern behavior at these parameters shows the insertions of reactant peaks.
(MOV)

Movie S2 Branching profile of first primary leaflet of RD BPM model. Ring was grown by pattern dependently in this simulation. One of the first primary leaflets was picked up for the observation of branch formation. Generated branches crossed but were independent to each other.
(MOV)

Movie S3 Branch formation by expansion inhibition. Constant expansion of the ring was inhibited in a pattern dependent manner in this simulation. Left panel shows the growth profile of the ring. First, second, and third splittings are marked by arrows. Right panel shows the spatiotemporal plot of the growing reaction-diffusion domain. Pattern behavior at these parameters shows the splittings of reactant peaks.
(MOV)
